# Transcriptional Regulation of Cysteine and Methionine Metabolism in *Lactobacillus paracasei* FAM18149

**DOI:** 10.3389/fmicb.2018.01261

**Published:** 2018-06-11

**Authors:** Daniel Wüthrich, Claudia Wenzel, Tharmatha Bavan, Rémy Bruggmann, Hélène Berthoud, Stefan Irmler

**Affiliations:** ^1^Interfaculty Bioinformatics Unit and Swiss Institute of Bioinformatics, University of Bern, Bern, Switzerland; ^2^Agroscope, Bern, Switzerland

**Keywords:** *Lactobacillus paracasei*, sulfur amino acid metabolism, cysteine, methionine, RNA-seq, differential gene expression

## Abstract

*Lactobacillus paracasei* is common in the non-starter lactic acid bacteria (LAB) community of raw milk cheeses. This species can significantly contribute to flavor formation through amino acid metabolism. In this study, the DNA and RNA of *L. paracasei* FAM18149 were sequenced using next-generation sequencing technologies to reconstruct the metabolism of the sulfur-containing amino acids cysteine and methionine. Twenty-three genes were found to be involved in cysteine biosynthesis, the conversion of cysteine to methionine and *vice versa*, the S-adenosylmethionine recycling pathway, and the transport of sulfur-containing amino acids. Additionally, six methionine-specific T-boxes and one cysteine-specific T-box were found. Five of these were located upstream of genes encoding transporter functions. RNA-seq analysis and reverse-transcription quantitative polymerase reaction assays showed that expression of genes located downstream of these T-boxes was affected by the absence of either cysteine or methionine. Remarkably, the *cysK2-ctl1-cysE2* operon, which is associated with te methionine-to-cysteine conversion and is upregulated in the absence of cysteine, showed high read coverage in the 5′-untranslated region and an antisense-RNA in the 3′-untranslated region. This indicates that this operon is regulated by the combination of *cis*- and antisense-mediated regulation mechanisms. The results of this study may help in the selection of *L. paracasei* strains to control sulfuric flavor formation in cheese.

## Introduction

Lactic acid bacteria (LAB), which can ferment diverse materials such as milk, meat, and plants, are widely used in food fermentation. *Lactobacillus paracasei* is of particular interest as it is found in a variety of habitats, including the human body, and fermented food. It is often found in cheese at the end of ripening, and the use of this species in adjunct cultures can improve ripening and flavor development (Beresford and Williams, 2004).

Volatile sulfur compounds (VSCs) are key flavor compounds in cheese mainly derived from microbial metabolism of sulfur-containing amino acids (Landaud et al., 2008). Various strains of *L. paracasei* produce VSCs when incubated with amino acids *in vitro* (Irmler et al., 2006). The production of VSCs is probably associated with side activities of enzymes involved in cysteine and methionine biosynthesis. This hypothesis is supported by the observation that the recombinant produced cysteine synthase CysK and the C-S lyases MalY, MetC and Ctl1 of *L. paracasei* produce hydrogen sulfide from cysteine and/or homocysteine (Irmler et al., 2008, 2009; Bogicevic et al., 2012a). Moreover, MetC and Ctl1 release methanethiol from methionine. Consequently, these enzymes could play a role in VSC formation in cheese. However, it is not known whether these genes are expressed in a cheese environment. A deeper understanding of the regulation of these genes can be helpful for a rational selection of strains to control VSC formation in cheese.

Comparative genomics has been used to identify the regulation mechanisms of cysteine and methionine biosynthesis in *Bacillales, Lactobacillales, Lactococcaceae*, and *Streptocococcaceae*. Regulation in streptococci is predicted to involve DNA-dependent systems and transcription factors of the LysR family (Rodionov et al., 2004; Kovaleva and Gelfand, 2007). Sulfur-metabolism genes in *Lactococcus lactis* are also regulated by a LysR-type transcription factor (Sperandio et al., 2005). In contrast, genes involved in methionine biosynthesis and transport in lactobacilli are predicted to be regulated by T-box leader sequences, which are *cis*-acting RNA regulatory elements that interact with tRNAs (Rodionov et al., 2004; Gutiérrez-Preciado et al., 2009; Liu et al., 2012). The binding of the tRNA molecule results in a change in the secondary structure of the leader sequence, which determines whether downstream genes will be expressed. A similar regulatory mechanism show S-box regulons (Grundy and Henkin, 1998), which are conserved RNA motifs that bind S-adenosylmethionine (SAM). A SAM-responsive regulatory element has been identified upstream of *metK*, which encodes SAM synthetase, in various LAB (Fuchs et al., 2006). The S-box regulon is the key regulator of cysteine and methionine metabolism in *Bacillus* and *Clostridium* organisms (Grundy and Henkin, 1998).

A previous study has uncovered that the expression of the *cysK2-ctl1-cysE2* gene cluster involved in the conversion of methionine to cysteine in *L. paracasei* FAM18149 was repressed in the presence of cysteine (Bogicevic et al., 2012b). This implied that cysteine may be an effector molecule that modulates gene expression. The present study extends to the overall response of *L. paracasei* FAM18149 to cysteine and methionine using next-generation sequencing technologies, bioinformatic analysis, and PCR-based methods. The expression of genes involved in cysteine and methionine metabolism of *L. paracasei* FAM18149 grown in a chemically defined medium (CDM) with cysteine as the sole sulfur source was compared to that of cells grown in CDM with methionine as the sole sulfur source.

## Material and methods

### Bacterial strain, media, and growth conditions

*Lactobacillus paracasei* FAM18149 was obtained from the Agroscope culture collection in Liebefeld (Bern, Switzerland). The strain was stored at −80°C in 10% sterile reconstituted skim milk powder and maintained at 30°C in MRS broth (De Man et al., 1960).

### Pacbio sequencing, genome assembly, and annotation

Genomic DNA (gDNA) was extracted from *L. paracasei* FAM18149 using the EZ1 DNA Tissue Kit (Qiagen, Hombrechtikon, Switzerland) according to the manufacturer's instructions. Before extraction, the bacterial cells were first treated with 0.1 M sodium hydroxide for 15 min at room temperature and then with lysozyme (50 mg dissolved in 0.1 M Tris[hydroxymethyl]-aminomethane, 10 mM ethylenediaminetetraacetic acid, 25% [w/v] sucrose, pH 8.0) for 1 h at 37°C.

The gDNA was sheared in a Covaris g-TUBE (Covaris, Woburn, MA, USA) to obtain 20-kb fragments and the size distribution of fragmented gDNA was analyzed on a Fragment Analyzer (Advanced Analytical Technologies, Ames, IA, USA). The sheared gDNA (5 μg) was used to prepare a SMRTbell library with the PacBio SMRTbell Template Prep Kit 1 (Pacific Biosciences, Menlo Park, CA, USA) according to the manufacturer's recommendations. The resulting library was size-selected on a BluePippin system (Sage Science, Inc. Beverly, MA, USA) for molecules larger than 14 kb. The recovered library was sequenced on one SMRT cell with P6/C4 chemistry and MagBeads on a PacBio RSII system (Pacific Biosciences, Menlo Park, CA, USA) at a 240-min movie length. Sequencing yielded 101,520 reads corresponding to 1,405 Mb with a mean read length of 13,840 bases. The resulting reads were assembled using the HGAP 3 (SMRT Analysis v-2.2.0) standard procedure (Chin et al., 2013). All scaffolds were annotated using the NCBI Prokaryotic Genome Annotation Pipeline. Coding sequences (CDSs) of interest were also searched against GenBank, and, in case of putative transporters, against the Transporter Classification Database (Saier et al., 2014).

### Detection of regulatory sequences

T-box leader sequences were identified with Infernal (version 1.1rc4) (Nawrocki and Eddy, 2013), which is included in the Prokka pipeline (Seemann, 2014). To identify methionine-specific T-box leader sequences, the T-box leader sequences were aligned to methionine-specific T-boxes of the *yxjH* gene from *Lactobacillus rhamnosus* (Lebeer et al., 2007). To identify cysteine-specific T-box leader sequences, T-boxes were aligned to cysteine-specific T-box leader sequences preceding *ubiG* (cpe0175), *cysP1* (cpe0947), *cysP2* (cpe0967), and *cysK* (cpe1322) of *Clostridium perfringens* strain 13 (André et al., 2010). The Clustal Omega algorithm was used for the alignments (Sievers et al., 2011). The conserved structural elements, including AGTA box, GNTG box, F-box, T-box, and specifier codons were identified based on the annotation of the aforementioned leader sequences. The S_MK_ box was identified by aligning the sequence described by Fuchs et al. (2006). Additional analyses were performed using RegRNA 2.0 (Chang et al., 2013).

### RNA isolation, rRNA depletion, and RNA sequencing (RNA-seq)

*L. paracasei* FAM18149 was grown in a chemically defined medium (CDM) described for *L. helveticus* (Christensen and Steele, 2003), omitting either cysteine (cysteine-deficient CDM) or methionine (methionine-deficient CDM) for 25 h at 30°C. The optical density of the culture was determined at 600 nm (OD600) with a spectrophotometer (LKB Biochrom 4050 Ultrospec II). An aliquot of approximately 10 OD was collected by centrifugation (3000 g, 10 min). RNA was isolated using the TRIzol®Max™ Bacterial RNA Isolation Kit (Life Technologies, Zug, Switzerland). Instead of a conventional phase separation, Direct-zol™RNA MiniPrep centrifugation columns (Zymo Research Corp., Irvine, USA) were used according to the manufacturer's instructions. For rRNA depletion, 1 μg of total RNA was used with the RiboMinus^TM^ Eukaryote System v2 protocol (ThermoFisher Scientific, Zug, Switzerland). The RiboMinus^TM^ Eukaryote probes provided by the kit were replaced by bacterial probes containing two 16S rRNA and three 23S rRNA sequence-specific 5′-biotin-labeled oligonucleotides, which were provided by ThermoFisher Scientific (Chen and Duan, 2011). Barcoded cDNA libraries were prepared using the IonExpress^TM^ RNA-Seq Barcode 1-16 Kit and the Ion Total RNA-seq v2 Kit (ThermoFisher Scientific). Two barcoded libraries were pooled and used for template preparation and enrichment with the Ion OneTouch™ 200 Template Kit v2 (ThermoFisher Scientific). Sequencing was performed on the Ion PGM™ using the Ion 318™ Chip and Ion PGM™ 200 Sequencing v2 Kits (ThermoFisher Scientific). The RNA was isolated from three biological repeats.

### Differential expression analysis

Reads shorter than 20 bp were removed using Trimmomatic version 0.36 (Bolger et al., 2014). Remaining reads were mapped to the genome assembly of *L. paracasei* FAM18149 (GenBank: GCA_002442835.1) using Bowtie2 (version 2.2.1, default parameters) (Langmead and Salzberg, 2012). For the differential expression analysis, the reads were counted using HTSeq (Anders et al., 2014), and only the reads that aligned to a predicted CDS were included (version 0.6.1, options: -a 1 -m intersection-nonempty). Finally, the dataset was normalized (size factor normalization) and tested (Wald test) for differential gene expression using DEseq2 (version 1.6.3) (Love et al., 2014). The resulting *p*-values were corrected for multiple testing using the Benjamini-Hochberg approach (Benjamini and Hochberg, 1995).

### Analysis of transcriptional organization

To visualize the transcriptional organization of significantly differentially expressed genes, the reads of all RNA-seq data sets were combined and aligned to the complete genome assembly of *L. paracasei* FAM18149 using Bowtie2 (version 2.2.1, default parameters). The read depth was determined using the Genome Analysis ToolKit (version 3.3.0) (McKenna et al., 2010) and plots for gene regions of interest were created with ggplot2 (Wickham, 2009).

### Gene set enrichment analysis

The CDSs of strain FAM18149 were searched against the SWISSPROT database (March 2017) using BLAST (version 2.2.31+), and against the Pfam database (March 2017) using pfam_search (version 1.6). The Gene Ontology (GO) terms obtained by these searches were assigned to the respective CDSs. The GO enrichment analysis was performed by comparing the genes that showed an adjusted *p*-value of < 0.05 on the differential expression analysis to the rest of the genome as background using the elim method of topGO (Alexa and Rahnenführer, 2018). All GO terms that had a *p*-value of ≤ 0.01 were considered to be significantly enriched.

### Reverse-transcription quantitative polymerase chain reaction (RT-qPCR)

*Lactobacillus paracasei* FAM18149 that had been grown for 25 h at 30°C either in cysteine-deficient or methionine-deficient CDM was collected by centrifugation. The cells were disrupted with zirconium beads (100 μm; OPS Diagnostics, Lebanon) using an Omni Bead Ruptor (6 ms^−1^ for 30 s; Labforce AG, Muttenz, Switzerland) and the RNA was isolated using the NucleoSpin® RNA plus Kit (Macherey-Nagel, Oensingen, Switzerland) according to manufacturer's instructions. To remove residual DNA, RNA samples were treated with DNaseI (Zymo Research Corp., Irvine, USA) according to the manufacturer's protocol. The RNA concentration was determined using the Qubit RNA BR Assay Kit (ThermoFisher Scientific). Total RNA (260 ng) was then used for single-stranded cDNA synthesis using the High Capacity cDNA Reverse Transcription Kit (Applied Biosystems, Foster City, USA) according to the manufacturer's protocol.

QPCR was carried out using a MIC qPCR Cycler (Bio Molecular Systems, Upper Coomera, Australia). The PCR reactions for *ctl1, cysK*, and *recA* contained hydrolysis probes. The PCR reactions (12 μL) contained Takyon No Rox Probe MaterMix UNG (Eurogentec, Seraing, Belgium), forward primer, reverse primer, hydrolysis probe, and 2 μL of cDNA that had been diluted in water (1:25). The primer and probe concentrations for *ctl1* and *cysK* were as described previously (Bogicevic et al., 2012a,b). For *recA*, 300 nM of recA_F, 300 nM of recA_R, and 100 nM of recA_FAM_probe were used. The other PCR reactions (12 μL) were based on DNA-binding dyes and contained Takyon No Rox SYBR MasterMix blue dTTP (Eurogentec), 300 nM of forward primer, 300 nM of reverse primer, and 2 μL of single-stranded cDNA that had been diluted in water (1:25). The primer sequences are listed in Table 1. The amplification conditions were 2 min at 50°C, 3 min at 95°C, followed by 40 cycles of 2 s at 95°C and 20 s at 60°C.

**Table 1:** Sequences of primers and fluorogenic probes used in this study.

**Primer**	**Sequence (5^′^-3^′^)**	**Target**	**Source or references**
metQ1_F	CAAGCCAATCTGAAGCACTTAAAG	*metQ1*	This study
metQ1_R	ATCAGTTCTGCGTCCAAATCG		
cysKI_F	CCGGCGGTTCTGTCAAAG	*cysK*	Bogicevic et al., 2012a
cysKI_R	CCCTTGTATTCGGCATCTTCA		
cysKI_FAM_probe	CCGAATTGCCTTGGCCATG		
metC_F	GCAGCGTCAAGGCTATTAGCA	*metC*	This study
metC_R	AGGAACGTGTTATCGACGATTGT		
yckJ_F	TTGGTTCTGCGTGAAATTATCATT	*yckJ*	This study
yckJ_R	CCTTGACGAGACTGATAAAACTGTTT		
metE_F	TGGCCTCAAGACTCGTGATG	*metE*	This study
metE_R	CCGCTGCCACCATGTTG		
yxjH_F	ACTTGGCTTCAAAGCTGTGACA	*yxjH*	This study
yxjH_R	AACCGTTCAAGCCCCATAAA		
metK_F	GCAAGCCGACTCTGGTTTG	*metK*	This study
metK_R	AAAAGGCACCACCACCATGT		
metQ3_F	GATATCGGCGCAACCTACATC	*metQ3*	This study
metQ3_R	TCGCCGTTCTTAACGTCCTT		
Q-ctl1_F	GCACTGGAAAGCTTGATCGAA	*ctl1*	Bogicevic et al., 2012b
Q-ctl1_R	ACCGAATGTCACGTGGAATTG		
Q-ctl1_FAM_probe	CGGCCTTGATGACCCACGGC		
glnP2_F	TACGCGTTTCCAGTGATCGG	*glnP2*	This study
glnP2_R	TGTAACTGCCGCCGAGAAAT		
glnM2_F	CAGAATTGTGCCAACCGTCG	*glnM2*	This study
glnM2_R	CAAGGTGGCCAATTGATCGC		
artM2_F	AACAGGACAAGTTCGCTGCT	*artM2*	This study
artM2_R	GGACAGTTGCCGGGGATATG		
glnH4_F	ATAAGGCGATCACCAAGGCC	*glnH4*	This study
glnH4_R	AGGCTGCTTGGACGTGAAAT		
recA_F	TTATGCGAATGGGTGCTAAGG	*recA*	This study
recA_R	CCAACACCAAGTGCATCATCA		
recA_FAM _probe	CGTTTCCGTTGTCTCTAGCGGCTCACT		
rpoB_F	GCTGAGCACACACGGGAAAT	*rpoB*	This study
rpoB_R	CAACTGCCACACTGGAAGCA		

All qPCR reactions were done in triplicates. Water was used as control. The crossing threshold cycle (Cq) was determined using the micPCR software v 2.4.0. The experiments were repeated three times.

## Results

### Reconstruction of cysteine and methionine metabolism

The assembly of the PacBio reads obtained from the sequencing of gDNA from *L. paracasei* FAM18149 resulted in six circular scaffolds (Supplementary Table S1). The largest scaffold, which represents the chromosome, comprised 2.7 Mbp whereas the plasmid scaffolds ranged from 28 to 84 kbp. The average read depth analysis indicated that three of the five plasmids were present in lower copy numbers.

Since the FAM18149 strain can grow in medium containing either sulfide, cysteine, or methionine as the sole sulfur source (Irmler et al., 2008; Bogicevic et al., 2016), the pathways for the conversion of cysteine to methionine and *vice versa* should be present. The FAM18149 genome was therefore searched for genes involved in the metabolism of these sulfur-containing amino acids. For readability purposes, the gene names are used in this report. The respective locus_tags are given in Table 2.

**Table 2:** DESeq2 results for genes associated with the metabolism of sulfur-containing amino acids in *L. paracasei* FAM18149.

**Gene (locus_tag)^a^**	**Function^b^**	**Induction methionine-deficient vs. cysteine-deficient CDM (log2 fold change)**	**Adjusted*p*-value**	**Transcriptional unit (regulatory element)**
*metQ1* (00255)	MULTISPECIES: substrate-binding protein of an ABC superfamily methionine transporter	−0.1	1.0	*metQ1* (T-box_met_)
*cysE* (00525)	serine O-acteyltransferase^c^	−1.2	4.1E-08	*cysE-cysK* (n. d.)
*cysK* (00530)	MULTISPECIES: cysteine synthase A	−1.1	1.5E-09	*cysE-cysK* (n. d.)
*metC* (00830)	cystathionine gamma-lyase^d^	1.5	5.7E-07	*yckJ-yckK-metC* (T-box_met_)
*yckK* (00835)	MULTISPECIES: amino acid ABC transporter substrate-binding protein	1.3	2.6E-04	*yckJ-yckK-metC* (T-box_met_)
*yckJ* (00840)	MULTISPECIES: cysteine ABC transporter permease	0.7	3.3E-01	*yckJ-yckK-metC* (T-box_met_)
*metF* (00930)	MULTISPECIES: 5,10-methylenetetrahydrofolate reductase	2.2	5.5E-19	*metE-metF* (T-box_met_)
*metE* (00935)	MULTISPECIES: 5-methyltetrahydropteroyltriglutamate–homocysteine S-methyltransferase	2.5	4.0E-36	*metE-metF* (T-box_met_)
*yxjH* (01620)	MULTISPECIES: vitamin-B12 independent methionine synthase	1.3	3.9E-07	*yxjH-luxS* (T-box_met_)
*luxS* (01625)	MULTISPECIES: S-ribosylhomocysteine lyase / autoinducer-2 production protein LuxS	0.4	7.2E-01	*yxjH-luxS* (T-box_met_)^e^
*malY* (02475)	MULTISPECIES: putative C-S lyase	0.3	0.7	*malY* (n. a.)
*metK* (02170)	MULTISPECIES: methionine adenoysltransferase	0.2	1.0	*metK* (S_MK_ box)
*metQ2* (04115)	MULTISPECIES: MetQ/NlpA family ABC transporter substrate-binding protein	0.7	1.0	*metQ2* (T-box_met_)
*metQ3* (04120)	MULTISPECIES: MetQ/NlpA family ABC transporter substrate-binding protein	1.2	5.4E-08	*metQ3-metN-metP* (T-box_met_)
*metN* (04125)	MULTISPECIES: ATP-binding protein of an ABC superfamily methionine transporter	1.1	9.9E-08	*metQ3-metN-metP* (T-box_met_)
*metP* (04130)	MULTISPECIES: ABC transporter permease	1.0	7.9E-04	me*tQ3-metN-metP* (T-box_met_)
*pfs* (04610)	5'-methylthioadenosine/S-adenosylhomocysteine nucleosidase	−0.3	0.9	*pfs* (n. a.)
*glnP2* (14340)	MULTISPECIES: polar amino acid ABC transporter inner membrane subunit	−2.5	1.6E-19	*glnP2-glnM2-artM2-glnH4* (T-box_cys_)
*glnM2* (14345)	MULTISPECIES: amino acid ABC transporter permease	−2.5	3.5E-17	*glnP2-glnM2-artM2-glnH4* (T-box_cys_)
*artM2* (14350)	MULTISPECIES: polar amino acid transport system ATP-binding protein	−2.6	1.5E-21	*glnP2-glnM2-artM2-glnH4* (T-box_cys_)
*glnH4* (14355)	MULTISPECIES: amino acid ABC transporter substrate-binding protein	−2.9	1.6E-33	*glnP2-glnM2-artM2-glnH4* (T-box_cys_)
*cysE2* (14655)	serine acetyltransferase	−2.9	2.0E-22	*cysE2-ctl1-cysK2* (RIT, antisense RNA)
*ctl1* (14660)	cystathionine beta and gamma-lyase	−3.2	8.3E-42	*cysE2-ctl1-cysK2* (RIT, antisense RNA)
*cysK2* (14665)	MULTISPECIES: cystathionine beta-synthase	−3.1	5.2E-34	*cysE2-ctl1-cysK2* (RIT, antisense RNA)

n. d, not detected; n. a, not analyzed; RIT, rho-independent terminator.

a Common gene names from Bacillus subtilis and Escherichia coli are used. Gene names for transporter are based on searches of the Transporter Classification Database (Saier et al., 2014). Numbers in brackets represent the locus_tag, of which the prefix FAM18149_ was omitted.

b Functions assigned based on BLAST searches.

c The gene name is designated as metA in L. paracasei ATCC 334. Since the gene in fact encodes a serine acetyltransferase (Bogicevic et al., 2016), the gene name cysE is used in this study.

d The recombinant-produced protein also exhibited cystathionine gamma-synthase activity in vitro (Irmler et al., 2008).

e The putative promoter 5′-TTAACA-N_18_-TATGAT-3′, which was identified in Lactobacillus rhamnosus GG (Lebeer et al., 2007), is also located upstream of luxS in L. paracasei FAM18149, indicating a T-box-independent expression of the respective gene.

**Figure 1:**
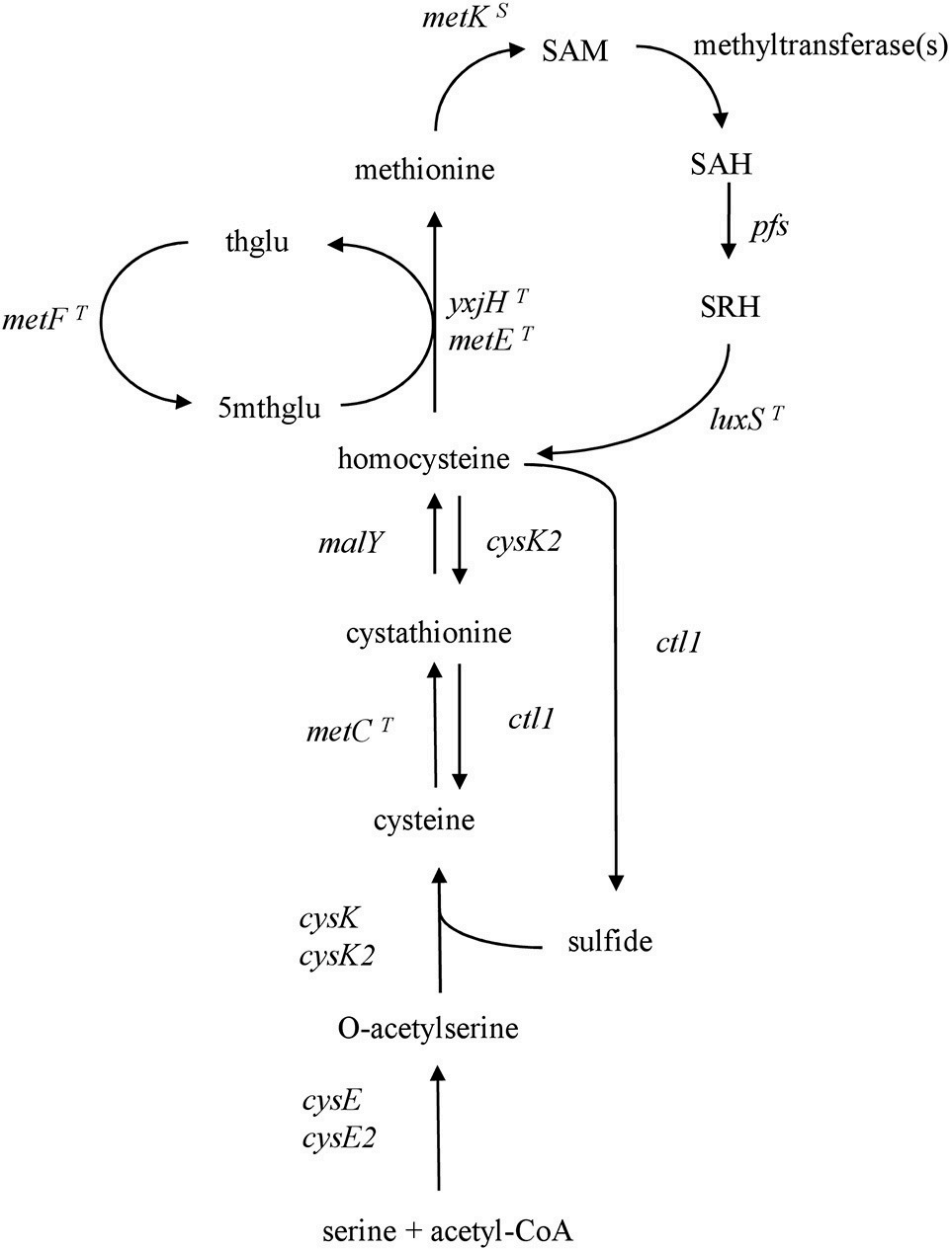
Reconstruction of cysteine and methionine metabolism in *L. paracasei* FAM18149. The gene names corresponding to locus-tags are presented in Table 2. A *T* or *S* in superscript indicates that the respective gene is probably regulated, respectively, by a T- or S-box regulation mechanism. thglu: tetrahydrofolate, 5mthglu: N5-methyl-tetrahydrofolate, SAM, S-adenosylmethionine; SAH, S-adenosylhomocysteine; SRH, S-ribosylhomocysteine.

A scheme of the reconstructed pathways is illustrated in Figure 1. *CysE* and *cysK* encode the enzymes that use serine, acetyl-CoA, and sulfide to produce cysteine. *MetC* and *malY* encode enzymes that form the transsulfuration pathway that converts cysteine to homocysteine. *CysK2, ctl1*, and *cysE2* are predicted to encode cystathionine beta-synthase, cystathionine lyase, and serine acetyltransferase, respectively, which are related to the conversion of methionine to cysteine. Based on these putative functions and the ability of recombinant Ctl1 to release sulfide from homocysteine, two pathways are proposed. In one, the sulfide produced by Ctl1 activity is used by CysE2 and CysK2 for cysteine biosynthesis. In the other, CysK2 could encode a cystathionine beta-synthase that synthesizes cystathionine from homocysteine, and Ctl1 subsequently cleaves cystathionine into cysteine and homoserine.

For methionine synthesis from homocysteine, the vitamin-B12 independent methionine synthases MetE and YxjH were identified. The methylation of homocysteine to yield methionine requires 5-methyltetrahydrofolate, which is provided by MetF activity. Methionine is used by MetK for SAM synthesis, the universal methyl donor. The latter compound is regenerated to methionine via the SAM recycling pathway, which involves methyltransferases, Pfs, and LuxS.

**Figure 2:**
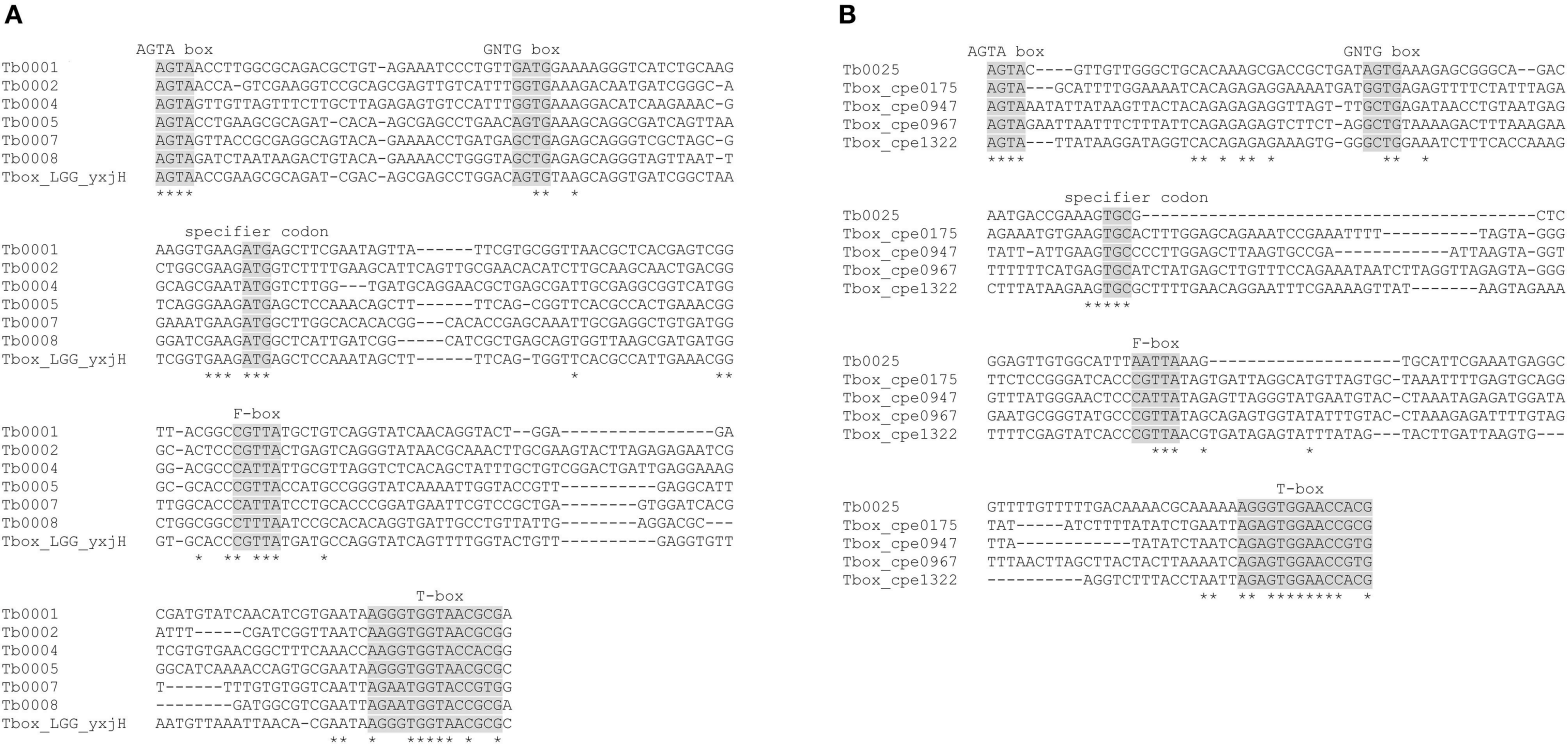
Alignment of methionine-specific T-boxes **(A)** and cysteine-specific T-boxes **(B)** of *L. paracasei* FAM18149 with T-boxes of *L. rhamnosus* (LGG_yxjH) and *C. perfringens* (cpe0175, cpe0947, cpe0967, and cpe1322). The conserved structural motifs (AGTA box, GNTG box, specifier codon, F-box, and T-box) are highlighted in gray. Invariant nucleotide sites are marked with asteriks.

In addition, a search was performed for T-boxes known to be modulated by cysteine or methionine. A total of 25 T-boxes were detected, of which 24 were located on the chromosome and one on a plasmid (Supplementary Table S2). By aligning the T-box sequences to the methionine-specific T-box preceding *yxjH* of *L. rhamnosus* GG, six T-Box leader sequences with the specifier codon ATG were identified (Figure 2A). These T-boxes were upstream of CDSs, encoding ABC transporter substrate-binding proteins (locus-tags FAM18149_00255, FAM18149_00840, FAM18149_04115, and FAM18149_04120), MetE and YxjH. With regard to cysteine-specific T-boxes, the specifier codon TGC was identified in in a T-box located on a plasmid (Figure 2B). The T-box preceded a gene encoding an amino acid ABC-transporter inner-membrane subunit (locus_tag FAM18149_14340). To label the transporter encoding CDSs with gene names, the deduced amino acid sequences were analyzed using the Transporter Classification Database (Saier et al., 2014). Based on the outcome of this search the locus_tags were given gene names as indicated in Table 2. With regard to S-box elements, the S_MK_ regulon that has previously been described for *L. casei* (Fuchs et al., 2006) was present in the 5′-untranslated region (UTR) of the *metK* gene (data not shown).

### Differential expression analysis

RNA from *L. paracasei* FAM18149 that had been grown in the absence of either cysteine or methionine was sequenced using semiconductor sequencing technology. The number of reads obtained from repeated transcriptomes of this strain varied from 921,890 to 3,126,855 (Supplementary Table S3), of which an average of 7.0% displayed an alignment quality of < 1 (low quality). Although the rRNA depletion step was applied, an average of 43% of the reads still mapped to rRNA genes and another 9.9% were aligned to tRNAs. Of the remaining reads, an average of 12.1 and 18.0%, respectively, mapped to non-coding features and CDSs of the *L. paracasei* FAM18149 genome.

Differential expression analysis using the DEseq2 algorithm identified 60 significantly (adjusted *p*-value < 0.05) differentially expressed protein-coding loci (Supplementary Table S4). With regard to the 24 genes predicted from the genomic data to be involved in cysteine and methionine metabolism (Table 2), nine genes (*cysE, cysK, glnP2, glnM2, artM2, glnH4, cysE2, clt1*, and *cysK2*) had higher expression in the cysteine-deficient medium, while eight others (*metC, yckK, metF, metE, yxjH, metQ2, metN*, and *metP*) had higher expression in methionine-deficient medium. It is noteworthy, that *metQ1* and *metQ2*, which are located downstream of two methionine-specific T-boxes, did not show significant differential regulation according to the DESeq2 analysis. A gene set enrichment analysis was also performed on the 60 significantly regulated genes, and nine GO terms were found that belonged to cysteine and methionine metabolism (Supplementary Table S5).

### Transcriptional organization

**Figure 3:**
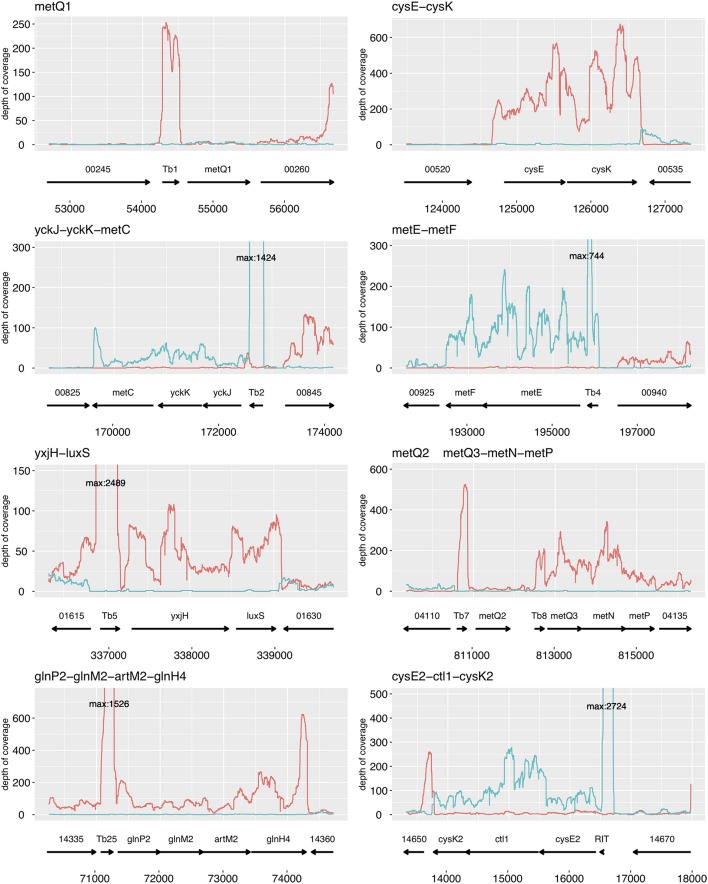
Identification of operons that are involved in cysteine/ methionine biosynthesis and transport in *L. paracasei* FAM18149. The plots illustrate the sequence coverage per base obtained from six RNA-seq datasets. The orientation of the sequence reads is represented in red (forward) and blue (reverse). The arrows represent genes and T-boxes. The numbers below the arrows indicate the genomic position.

To identify operons, all six RNA-seq datasets were mapped onto the corresponding regions including the upstream and downstream loci (Figure 3). Since the strand information was retained, the reads could be assigned to the direction of transcription relative to the sense or antisense information of the loci. This process identified the cotranscription of the following genes: *cysE-cysK, yckJ-yckK-metC, metE-metF, yxjH-luxS, metQ3-metN-metP, glnP2-glnM2-artM2-glnH4*, and *cysE2-ctl1-cysK2*. Remarkably, the T-box regions displayed considerable high coverage (Figure 3). High coverage of the 5′-UTR was also oberserved for the *cysK2-ctl1-cysE2* operon, and antisense-RNA was found in the 3′-UTR of this operon (Figure 3).

### Targeted gene expression analysis using RT-qPCR

The expression of 13 genes associated with the metabolism of sulfur-containing amino acids was analyzed using RT-qPCR as an alternative method (Table 3). The *recA* and *rpoB* genes, encoding the recombinase recA and the beta subunit of the DNA-directed RNA polymerase, respectively, were used as “constitutively” expressed control genes. The selected genes confirmed the results obtained from the DESeq2 analysis except for *metQ1* and *cysK*. *MetQ1* had slightly higher expression in the methionine-deficient medium compared to the cysteine-deficient medium, while no significant differential expression was found for *cysK*.

**Table 3:** Targeted gene expression analysis using RT-qPCR.

**Gene (locus tag)^a^**	**Cysteine-deficient CDM**	**Methionine-deficient CDM**
*recA* (02620)	23.8 ± 0.6	23.6 ± 0.6
*rpoB* (10445)	18.3 ± 0.9	19.3 ± 0.9
*metQ1* (00255)	25.5 ± 0.4	24.2 ± 0.5
*cysK* (00530)	22.3 ± 0.6	23.0 ± 0.8
*metC* (00830)	20.8 ± 0.4	18.0 ± 0.4
*yckJ* (00840)	24.6 ± 0.4	21.9 ± 0.5
*metE* (00935)	21.0 ± 0.4	17.7 ± 0.4
*yxjH* (01620)	22.1 ± 0.4	20.2 ± 0.8
*metK* (02170)	18.1 ± 1.1	18.2 ± 0.8
*metQ3* (04120)	21.3 ± 0.3	19.8 ± 0.6
*glnP2* (14340)	19.2 ± 0.3	22.9 ± 0.7
*glnM2* (14345)	19.3 ± 0.8	22.4 ± 0.5
*artM2* (14350)	18.7 ± 0.5	22.3 ± 0.5
*glnH4* (14355)	17.6 ± 0.4	21.0 ± 0.8
*ctl1* (14660)	21.4 ± 0.6	25.7 ± 0.6

The numbers represent the average of the quantification cycle value and standard deviation obtained from three independent experiments.

a Numbers in brackets represent the locus_tag, of which the prefix FAM18149_ was omitted.

## Discussion

Bacteria are known to synthesize the sulfur-containing amino acids cysteine and methionine from serine and homoserine, respectively. Furthermore, bacteria possess transport systems to take up amino acids from the environment. Cysteine and methionine biosynthesis has been well studied in *Escherichia coli*, in which the consecutive actions of serine acetyltransferase and O-acetylserine sulfhydrylase, which are respectively encoded by *cysE* and *cysK*, convert serine to cysteine (Kredich, 1996). Cysteine is then converted via cystathionine into methionine, which involves the activity of a cystathionine gamma-synthase (MetB) and a cystathionine beta-lyase (MetC). Bioinformatic analysis of the first published *L. paracasei* ATCC 334 (formerly *Lactobacillus casei*) genome indicated that this species does not possess orthologs of *cysE* and *metB* and could be auxotrophic for methionine (Makarova et al., 2006). In contrast, various *L. paracasei* strains including ATCC 334 grow in the absence of methionine (Irmler et al., 2008). By cloning and heterologously expressing genes that encode putative C-S lyases, it was found that the gene annotated as *metC* in strain ATCC 334 not only degraded but also synthesized cystathionine, depending on the *in vitro* conditions (Irmler et al., 2008). In addition, the gene annotated as *metA* in *L. paracasei* does not encode a homoserine O-succinyltransferase but does encode a serine acetyltransferase (Bogicevic et al., 2016). Thus, the majority of *L. paracasei* strains likely possess pathways for cysteine and methionine synthesis that are similar to those of *E. coli*. The genome analysis of strain FAM18149 also revealed the presence of all genes necessary for the SAM recycling.

Interestingly, few bacteria, including *Mycobacterium tuberculosis, Klebsiella pneumoniae, Bacillus subtilis*, and *Clostridium acetobutylicum*, possess a reverse transsulfuration pathway (Wheeler et al., 2005; Seiflein and Lawrence, 2006; Hullo et al., 2007; André et al., 2008). This pathway converts homocysteine to cysteine via the intermediate cystathionine, and requires a cystathionine beta-synthase (CBS) and a cystathionine gamma-lyase (CGL). Since *L. paracasei* FAM18149 can grow in cysteine-deficient medium, it is likely that it possesses the reverse transsulfuration pathway. Ctl1 could represent the cystathionine lyase needed for this pathway (Irmler et al., 2009). However, to the best of our knowledge, the required CBS activity has not yet been shown in *L. paracasei*. Consequently, it is proposed that as an alternative to the reverse transsulfuration pathway,the FAM18149 strain may break down homocysteine to release sulfide that is then used for cysteine biosynthesis. Figure 1 illustrates a schematic model of cysteine and methionine biosynthesis in *L. paracasei* FAM18149.

With regard to regulation mechanisms, six methionine-specific T-boxes and one cysteine-specific T-box were found. Rodionov et al. (2004) analyzed the unfinished genome of *L. paracasei* ATCC 334 and predicted methionine-specific T-boxes for *metE-metF, yxjH-luxS, metQ2*, and *metQ3NP*. Later, Gutiérrez-Preciado et al. (2009) identified a fifth methionine-specific T-box upstream of an operon designated as *glnH-glnH-yjcJ* in *L. casei*, which probably represents the *yckJK*-*metC* operon described in the present study. Five T-boxes of FAM18149 were located upstream of CDSs encoding transporter subunits. Identification of the specifier codon of T-boxes can be used to predict the substrate specificity of the downstream genes that encode transporters (Wels et al., 2008). Therefore, the genes *metQ1*, and *metQ2* and the operon *metQ3NP* can be associated with methionine transport. Since the *metQ3NP* operon also showed homology to the *metNPQ* transport system of *Bacillus subtilis* that transports L- and D-isomers of methionine as well as an oxidation product of methionine (Hullo et al., 2004), it is assumed that the gene products are actually involved in methionine import. *MetQ1* and *metQ2* are predicted to encode periplasmatic subunits of transport systems. Further studies are necessary to identify the transmembrane domains (TMDs) to which these bind.

The *yckJK-metC* operon is of special interest. BLAST searches of GenBank and the Transporter Classification Database indicated that the gene products of *yckJ* and *yckK* form a cystine/cysteine ABC transport system. However, the genes are regulated by a methionine-specific T-box, predicting methionine as substrate. Structural analysis of various bacterial ABC transporters revealed that ABC transport systems generally consist of periplasmatic subunits that capture the substrate, subunits with TMDs, and membrane-associated subunits that possess nucleotide-binding domains (NDBs) (Wilkens, 2015). NDBs bind and hydrolyze ATP which is necessary for the transport mechanism. With regard to *yckJK, metC* is present instead of an NDB subunit. Based on the observation that MetC hydrolyzed O-succinyl-L-homoserine *in vitro* (Irmler et al., 2009), it is thought that this hydrolysis could drive the transport. Therefore, we assume that *yckJK* actually transports cysteine, which is subsequently used by MetC for cystathionine synthesis, the precursor for methionine biosynthesis.

The presence of a cysteine-specific T-boxs upstream of the *glnP2-glnM2-artM2-glnH4* operon indicates that the gene products also form a cysteine ABC transport system. Sequence analysis of the CDSs encoded by this operon showed a relationship to glutamine/glutamate transporters. As the operon was more highly expressed in the absence of cysteine, it may indeed be involved in the transport of cysteine or of the precursors needed for cysteine biosynthesis. This transport system was also found on a plasmid in *L. paracasei* ATCC 334 (GenBank acc. no.CP000424) but not in other *L. paracasei* genomes in the GenBank database. Since cheese is a cysteine-poor environment, the presence of a cysteine transport system apparently does not provide an evolutionary advantage for this bacterium, which could explain why this transport system is not widely distributed in *L. paracasei* strains, especially those of dairy origin.

Analysis of the RNA-seq data enabled identification of the genes that formed transcriptional units (Figure 3). This made it possible to confirm that the *cysE-cysK* and *cysK2-ctl1-cysE2* gene clusters were transcribed as polycistronic RNAs, which had been previously demonstrated by PCR methods (Bogicevic et al., 2012a,b). Furthermore, the RNA-seq and the gene set enrichment analysis clearly showed that the expression of many genes involved in cysteine and methionine biosynthesis and transport respond to the availability of cysteine and methionine. With regard to the SAM recycling pathway, the expression levels for *pfs* and *luxS* did not show significant differences between cells grown in cysteine-deficient or methonine-deficient medium. In the closely related species *L. rhamnosus*, monocistronic, and polycistronic RNAs of *luxS* were identified (Lebeer et al., 2007). This may explain why this gene seems to be unaffected by cysteine or methionine, although it forms an operon with *yxjH*, which is controlled by a methionine-specific T-box.

RNA-seq analysis showed that the expression of genes located downstream of methionine- and cysteine-specific T-boxes, except for *metQ1* and *metQ2*, were affected by the absence of either cysteine or methionine. RT-qPCR analysis confirmed this observation. Additionally, RT-qPCR showed that the expression of *metQ1* was lower in the absence of methionine. The extremely low read coverage for this gene in the RNA-seq data set was likely the reason it was not determined by the DESeq2 algorithm to be significantly expressed.

Remarkably, all T-box leader sequences displayed extremely high read coverage (Figure 3). There are probably two main reasons for this finding. First, the T-boxes mediate the premature termination of transcription yielding more transcript sequence reads for the 5′-UTR than for the downstream located CDS regions. Second, RNase III, which was used in this study for RNA fragmentation, preferentially cleaves double-stranded RNA. T-boxes that form complex secondary structures (Vitreschak et al., 2008) are therefore probably prone to RNase III activity, which could subsequently lead to enrichment of T-boxes in the cDNA libraries.

The 5′-UTR of the *cysE-cysK* and *cysK2-ctl1-cysE2* operons did not contain T-box leader sequences. The DESeq2 analysis showed a small difference of expression for the *cysE-cysK* operon between the cysteine- and methionine-deficient media. In-depth analysis of the nucleotide sequence of the 5′-UTR did not provide hints of any regulation mechanisms (data not shown). When *cysK* expression was studied using RT-qPCR analysis, no significant difference was observed between the cysteine- and methionine-deficient media-grown cells. Therefore, it is assumed that the *cysE-cysK* operon was constitutively expressed in the present study.

The high read coverage in the 5′-UTR of the *cysK2-ctl1-cysE2* operon indicates a mechanism of regulation similar to that of S-box and T-box leader sequences. However, these regulons could not be identified. Liu et al. (2012) identified the palindromic motif AAAGGGCGCGAA-N_(11−18)_-TTCGCGCCTTTT as a potential regulatory motif upstream of genes encoding cystathionine synthase and cystathionine lyase in lactobacilli. In strain FAM18149, the similar motif AAGGGCGCGAAA-N_16_-TTTCGCGCCCTT can be found upstream of the *cysK2-ctl1-cysE2* operon. When the 5′-UTR was analyzed using RegRNA 2.0, a 60-bp rho-independent terminator region that covered the aforementioned motif was identified. The sequence of this region can base-pair with itself and form a stem-loop structure. This stem-loop structure could cause the RNA polymerase to stall or stop the transcription. The high read coverage present in the 5′-UTR of the *cysK2-ctl1-cysE2* operon supports the hypothesis that expression is regulated by premature termination of transcription. The occurrence of antisense RNA in the 3′-UTR of this operon could be an additional regulatory element. The combination of a *cis-* and antisense-mediated regulation was recently detected in *Clostridium acetobutylicum* (André et al., 2008). Interestingly, in this species it regulates the *ubiG* operon, which is also involved in methionine-to-cysteine conversion.

The present study showed that the genes involved in the metabolism of sulfur-containing amino acids in *L. paracasei* are regulated mainly at the transcriptional level. Methionine-specific T-box regulons regulate methionine biosynthesis and uptake, but the molecular mechanism of regulation for the expression of the *cysK2-ctl1-cysE2* operon involved in the methionine-to-cysteine conversion is yet to be discovered. The RNA-seq analysis in this study was a valuable tool for studying *cis*-mediated regulation, verifying operon structure, and identifying active and inactive genes. Combined with the enzymatic characterization, RNA-seq helps to reveal metabolic pathways used by bacteria species in cheese providing a clearer understanding of the biochemistry of cheese.

## Author contributions

SI and HB conceived and designed the study. SI and TB performed the RNA-seq experiments. DW, SI, and RB performed the bioinformatic analysis. HB and CW carried out the RT-qPCR analysis. SI, HB, DW, RB, and CW contributed to data interpretation. DW and SI wrote the manuscript. All authors approved the work for publication.

### Conflict of interest statement

The authors declare that the research was conducted in the absence of any commercial or financial relationships that could be construed as a potential conflict of interest.
