# Supplementary material for: Transcriptional Regulation of Cysteine and Methionine Metabolism in Lactobacillus paracasei FAM18149
Source: Front Microbiol. 2018 Jun 11;9:1261. doi: 10.3389/fmicb.2018.01261 (PMC6004538; doi:10.3389/fmicb.2018.01261)
Supplement: Supplementary file 1 [file Table_1.DOCX]

**Table S1** Genome statistics of the complete genome assembly of *Lactobacillus paracasei* FAM18149

|  | **Scaffold 1** | **Scaffold 2** | **Scaffold 3** | **Scaffold 4** | **Scaffold 5** | **Scaffold 6** |
| --- | --- | --- | --- | --- | --- | --- |
| GenBank acc. no. | CP017261 | CP017262 | CP017263 | CP017264 | CP017265 | CP017266 |
| Lenght (bp) | 2,710,941 | 62,971 | 36,931 | 83,229 | 28,557 | 47,078 |
| GC content (%) | 46.6 | 43.9 | 40.2 | 43.5 | 39.9 | 42.8 |
| Average read depth | 380.6 | 381.3 | 77.8 | 300.5 | 49.7 | 90.6 |
| rRNA operon copy number | 5 | 0 | 0 | 0 | 0 | 0 |
| tRNAs | 59 | 0 | 0 | 0 | 0 | 0 |
| CDSs | 2593 | 78 | 45 | 92 | 34 | 50 |
| locus_tag of replication initiation protein | FAM18149_06755  FAM18149_11905 | FAM18149_13645 | FAM18149_13895  FAM18149_13905 | FAM18149_14200  FAM18149_14260 | FAM18149_14585 | FAM18149_14266 |
| Pseudogenes (total) | 105 | 2 | 3 | 12 | 1 | 6 |
| Transposases | 140 | 15 | 13 | 15 | 2 | 23 |
| T-boxes | 24 | 0 | 0 | 1 | 0 | 0 |

bp: base pair, CDSs: protein-coding sequences
