# Supplementary material for: Transcriptional Regulation of Cysteine and Methionine Metabolism in Lactobacillus paracasei FAM18149
Source: Front Microbiol. 2018 Jun 11;9:1261. doi: 10.3389/fmicb.2018.01261 (PMC6004538; doi:10.3389/fmicb.2018.01261)
Supplement: Supplementary file 2 [file Table_2.DOCX]

**Table S2** T-boxes identified in *Lactobacillus paracasei* FAM18149

| **T-Box names** | **Sequence** | **Start** | **End** | **Strand** | **specificity** | **downstream located CDS (locus_tag FAM18149_)** |
| --- | --- | --- | --- | --- | --- | --- |
| Tb0001 | CP017261 | 54292 | 54518 | + | met | ABC transporter substrate-binding protein (00255) |
| Tb0002 | CP017261 | 172597 | 172838 | - | met | MULTISPECIES: cysteine ABC transporter permease (00840) |
| Tb0003 | CP017261 | 176094 | 176332 | - |  | branched-chain amino acid transport system II carrier protein (00850) |
| Tb0004 | CP017261 | 195840 | 196083 | - | met | 5-methyltetrahydropteroyltriglutamate-- homocysteine S-methyltransferase (00935) |
| Tb0005 | CP017261 | 336894 | 337126 | + | met | MULTISPECIES: vitamin-B12 independent methionine synthase (01620) |
| Tb0006 | CP017261 | 438346 | 438539 | + |  | leucine--tRNA ligase (02200) |
| Tb0007 | CP017261 | 810626 | 810858 | + | met | MULTISPECIES: MetQ/NlpA family ABC transporter substrate-binding protein (04115) |
| Tb0008 | CP017261 | 812522 | 812750 | + | met | MULTISPECIES: ABC transporter substrate-binding protein (04120) |
| Tb0009 | CP017261 | 874637 | 874837 | + |  | valine--tRNA ligase (04425) |
| Tb0010 | CP017261 | 884965 | 885190 | + |  | amino acid ABC transporter permease (04470) |
| Tb0011 | CP017261 | 903769 | 903974 | + |  | isoleucine--tRNA ligase (04570) |
| Tb0012 | CP017261 | 1061490 | 1061744 | - |  | ATP phosphoribosyltransferase regulatory subunit (05265) |
| Tb0013 | CP017261 | 1143658 | 1143824 | - |  | glycine--tRNA ligase subunit alpha (05605) |
| Tb0014 | CP017261 | 1190817 | 1190974 | - |  | histidine--tRNA ligase (05865) |
| Tb0015 | CP017261 | 1334604 | 1334820 | - |  | phenylalanine--tRNA ligase subunit alpha (06585) |
| Tb0016 | CP017261 | 1361514 | 1361711 | - |  | threonine--tRNA ligase (06745) |
| Tb0017 | CP017261 | 1388661 | 1388825 | - |  | arginine--tRNA ligase (06870) |
| Tb0018 | CP017261 | 1499284 | 1499473 | - |  | serine--tRNA ligase (07415) |
| Tb0019 | CP017261 | 1646875 | 1647064 | - |  | tyrosine--tRNA ligase (08200) |
| Tb0020 | CP017261 | 2225151 | 2225331 | - |  | tryptophan--tRNA ligase (10995) |
| Tb0021 | CP017261 | 2312233 | 2312501 | - |  | peptide ABC transpoter substrate-binding protein (11410) |
| Tb0022 | CP017261 | 2470369 | 2470648 | - |  | anthranilate phosphoribosyltransferase (12165) |
| Tb0023 | CP017261 | 2474640 | 2474920 | - |  | PTS maltose transporter subunit IIBC (12185) |
| Tb0024 | CP017261 | 2655115 | 2655359 | - |  | branched-chain amino acid transport system II carrier protein (13095) |
| Tb0025 | CP017264 | 71089 | 71278 | + | cys | MULTISPECIES: polar amino acid ABC transporter inner membrane subunit (14340) |
