# Supplementary material for: Transcriptional Regulation of Cysteine and Methionine Metabolism in Lactobacillus paracasei FAM18149
Source: Front Microbiol. 2018 Jun 11;9:1261. doi: 10.3389/fmicb.2018.01261 (PMC6004538; doi:10.3389/fmicb.2018.01261)
Supplement: Supplementary file 3 [file Table_3.DOCX]

**Table S3** General features of the RNA-seq data sets from *Lactobacillus paracasei* FAM18149

| **Feature** | **methionine-deficient CDM** | | | **cysteine-deficient CDM** | | |
| --- | --- | --- | --- | --- | --- | --- |
|  | replicate 1 | replicate 2 | replicate 3 | replicate 1 | replicate 2 | replicate 3 |
| Reads mapped to CDSs (%) | 20.75 | 15.67 | 18.06 | 22.05 | 14.19 | 17.11 |
| Reads mapped to rRNA coding genes (%) | 39.94 | 56.98 | 40.01 | 41.83 | 44.15 | 35.41 |
| Reads mapped to tRNA coding genes (%) | 11.16 | 3.94 | 12.49 | 7.89 | 6.29 | 17.51 |
| Reads mapped to the tmRNA coding gene (%) | 1.43 | 1.05 | 0.79 | 1.29 | 0.88 | 0.81 |
| Reads mapped to non-coding sequences (%) | 10.49 | 8.13 | 17.02 | 9.76 | 11.13 | 16.17 |
| Multiple features (%) | 0 | 0 | 0 | 0 | 0 | 0 |
| Reads of low quality (%) | 7.36 | 5.91 | 6.95 | 6.9 | 7.76 | 7.1 |
| Not aligned reads (%) | 8.87 | 8.31 | 4.68 | 10.27 | 15.6 | 5.89 |
| No. of reads | 921,890 | 1,263,141 | 2,124,561 | 970,041 | 983,926 | 3,126,855 |
| Average read length (bp) | 107.1 | 95.6 | 86.8 | 121.6 | 89.1 | 88.2 |

CDSs: protein-coding sequences; rRNA: ribosomal RNA, tRNA: transfer RNA tmRNA: transfer-messenger-RNA
