# Supplementary material for: Transcriptional Regulation of Cysteine and Methionine Metabolism in Lactobacillus paracasei FAM18149
Source: Front Microbiol. 2018 Jun 11;9:1261. doi: 10.3389/fmicb.2018.01261 (PMC6004538; doi:10.3389/fmicb.2018.01261)
Supplement: Supplementary file 5 [file Table_5.DOCX]

**Table S5** Gene set enrichment analysis using topGo.

| **GO.ID** | **Term** | **Annotated** | **Significant** | **Expected** | **Rank in Fischer.classic** | **Fischer.classic** | **Fischer.elim** |
| --- | --- | --- | --- | --- | --- | --- | --- |
| GO:0006535 | cysteine biosynthetic process from serine | 6 | 4 | 0.11 | 10 | 1.30E-06 | 1.30E-06 |
| GO:0015811 | L-cystine transport | 22 | 5 | 0.4 | 23 | 2.90E-05 | 2.90E-05 |
| GO:0019279 | L-methionine biosynthetic process from L-homoserine via cystathionine | 2 | 2 | 0.04 | 32 | 0.00032 | 0.00032 |
| GO:0042883 | cysteine transport | 23 | 4 | 0.42 | 34 | 0.00061 | 0.00061 |
| GO:0019343 | cysteine biosynthetic process via cystathionine | 3 | 2 | 0.05 | 37 | 0.00094 | 0.00094 |
| GO:0019346 | transsulfuration | 3 | 2 | 0.05 | 38 | 0.00094 | 0.00094 |
| GO:0050667 | homocysteine metabolic process | 7 | 4 | 0.13 | 14 | 3.00E-06 | 0.00162 |
| GO:0048473 | D-methionine transport | 4 | 2 | 0.07 | 40 | 0.00187 | 0.00187 |
| GO:0009086 | methionine biosynthetic process | 18 | 5 | 0.33 | 18 | 1.00E-05 | 0.00209 |
